# Supplementary material for: Advanced Anterior Eye Segment Imaging for Ichthyosis
Source: J Clin Med. 2023 Sep 16;12(18):6006. doi: 10.3390/jcm12186006 (PMC10532152; doi:10.3390/jcm12186006)
Supplement: Supplementary file 1 [file jcm-12-06006-s001.zip › jcm-2506154-supplementary.pdf]

Journal of Clinical Medicine

Table S1. Analyzed parameters of the anterior segment of the eye.

Parameter	Development of an English Abbreviation	Description of the Parameter	Unit
**Corneal Parameters [k, keratometric; r, real; p, posterior]**			
**Ks**	Keratometry steep	Steep meridian value of keratometry of the curvature of the cornea	[D]—Diopter
**Kf**	Keratometry flat	Flat meridian value of keratometry of the curvature of the cornea	[D]—Diopter
**CYL**	Cylinder	Astigmatism power	[D]—Diopter
**AvgK**	Average Keratometry	Mean value of steep meridian Ks and flat meridian Kf	[D]—Diopter
**Ecc**	Eccentricity corneal curve	Ecc corresponds to the numeric value of the eccentricity at a 9 mm diameter area. Eccentricity describes the rate of corneal flattening from the central cornea to the periphery	Numerical range 0-1
**AA [%]**	Area Analyzed	AA reflects the percentage of performed automatic analysis at a 10 mm diameter area [assuming the whole circumference as 100%]	[%] percentage
**ACCP**	Average Central Corneal Power	The mean value of axial power within a 3 mm diameter	[D]—Diopter
**CAT**	Central Apical Thickness	The corneal thickness on the measurement axis	[μm]—micrometers
**CTT**	Corneal Thinnest Thickness	Corneal thinnest thickness	[μm]—micrometers
**CAT-CTT**	Central Apical Thickness minus Corneal Thinnest Thickness	The difference between central apical thickness and corneal thinnest thickness	[μm]—micrometers
**ACD [mm]**	Anterior Chamber Depth	Anterior chamber depth from the posterior corneal surface to the anterior crystalline lens surface	[mm] millimeters
**ESI [%]**	Ectasia Screening Index	ESI (%) is assessed based on the following data: the thinnest corneal thickness (μm) and its location related to the corneal apex (mm) (X, Y coordinates); FI of the keratometric and posterior topography data; the location	[%] percentage

		and the lowest value of the instantaneous posterior power within a 6 mm diameter.	
**Fourier indices; area of 3 mm and 6 mm; k, keratometric; a, anterior; p, posterior**			
**Spherical**	Fourier Index Spherical	The spherical refractive power component of the cornea obtained by Fourier analysis of the topographic data of the cornea with a diameter of 3 and 6 mm. Corresponds to the zero-order component in Fourier analysis	[D]—Diopter
**Reg. Astigmatism**	Fourier Index Regular	The component of regular corneal refractive power astigmatism obtained by Fourier analysis of corneal topographic data with a diameter of 3 and 6 mm. Corresponds to the second-order component in the Fourier analysis	[D]—Diopter
**Asymmetry**	Fourier Index Asymmetry	Asymmetric refractive power component of the cornea obtained by Fourier analysis of corneal topographic data with a diameter of 3 and 6 mm. Corresponds to the first-order component in Fourier analysis	[D]—Diopter
**Higher Order**	Fourier Index Higher Order	Higher order irregularities—calculated by combining tertiary and higher components in Fourier analysis	[D]—Diopter

Table S2. BCVA, ocular surface examination, and corneal topography and thickness map results.								
Parameter	P1	P2	P3	P4	P5	min	max	median
BCVA	RE 0,8<br>LE 0,7	RE 0,2<br>LE LP	RE 0,05<br>LE 0,1	RE 0,3<br>LE 0,2	RE 1,0<br>LE 0,4	LP	1,0	0,25
TBUT [s]	RE 6<br>LE 8	RE 4<br>LE 4	RE 7<br>LE 8	RE 5<br>LE 5	RE 12<br>LE 8	4	10	6,5
Fluorescein staining [Oxforrd scale]	RE IV<br>LE II	RE III<br>LE IV	RE IV<br>LE III	RE II<br>LE II	RE 0<br>LE I	0	IV	
kKs [D]	RE 43,1<br>LE 42,0	RE 48,1<br>LE 68,6	RE 57,3<br>LE 53,4	RE 50,4<br>LE 47,5	RE 45,1<br>LE44,7	42	68,6	47,8
kKf [D]	RE 41,2<br>LE 41,5	RE 45,8<br>LE 62,8	RE 53,6<br>LE 46,3	RE 47,4<br>LE 46,4	RE 42,7<br>LE 42,2	41,2	62,8	46,05
kCYL [D]	RE 1,9<br>LE 0,5	RE 2,3<br>LE 6,2	RE 3,8<br>LE 7,1	RE 2,9<br>LE 1,2	RE 2,4<br>LE 2,5	0,5	7,1	2,45
kAvgK [D]	RE 42,1<br>LE 41,8	RE 47,0<br>LE 66,2	RE 55,4<br>LE 49,8	RE 48,9<br>LE 47,0	RE 43,9<br>LE 43,5	41,8	66,2	47
kEcc	RE 0,21<br>LE 0,32	RE 0,31<br>LE 0,32	RE 0,54<br>LE 0,55	RE 0,73<br>LE 0,61	RE 0,53<br>LE 0,59	0,21	0,73	0,53
kAA [%]	RE 97,2<br>LE 98,9	RE 94,0<br>LE 89	RE 100<br>LE 99,9	RE 97<br>LE 90	RE 93,3<br>LE 97,5	89	100	97,1
kACCP [D]	RE 42,1<br>LE 41,8	RE 47,4<br>LE 65,5	RE 55,7<br>LE 49,4	RE 49,4<br>LE 47,0	RE 43,9<br>LE 43,8	41,8	65,5	47,2
pKs [D]	RE -6,2<br>LE -6,3	RE -6,5<br>LE -11,1	RE -8,0<br>LE -6,8	RE -7,7<br>LE -6,9	RE -6,8<br>LE -6,7	-11,1	-6,2	-6,8
pKf [D]	RE -5,9<br>LE -6,0	RE -6,5<br>LE -9,3	RE -7,3<br>LE -6,3	RE -7,1<br>LE -6,5	RE -6,1<br>LE -6,0	-9,3	-5,9	-6,4
pCYL [D]	RE 0,3<br>LE 0,3	RE 0,1<br>LE 1,8	RE 0,7<br>LE 0,6	RE 0,6<br>LE 0,4	RE 0,7<br>LE 0,7	0,1	1,8	0,6
pAvgK [D]	RE -6,0<br>LE -6,2	RE -6,5<br>LE -10,1	RE -7,6<br>LE -6,5	RE -7,4<br>LE -6,7	RE -6,4<br>LE -6,3	-10,1	-6	-6,5

pEcc	RE 0,48<br>LE 0,65	RE 0,31<br>LE 0,83	RE 0,59<br>LE 0,57	RE 0,83<br>LE 0,65	RE 0,7<br>LE 0,6	0,31	0,83	0,63
pAA [%]	RE 94<br>LE 94,6	RE 91,4<br>LE 66,7	RE 100<br>LE 98,5	RE 94,5<br>LE 88,1	RE 90,7<br>LE 95,2	66,7	100	94,25
rKs [D]	RE 42,1<br>LE 40,9	RE 47,3<br>LE 65,4	RE 58,0<br>LE 52,9	RE 48,6<br>LE 46,2	RE 43,6<br>LE 43,3	40,9	65,4	46,75
rKf [D]	RE 40,0<br>LE 40,1	RE 44,9<br>LE 63,8	RE 52,6<br>LE 45,6	RE 46,0<br>LE 45,2	RE 41,6<br>LE 41,1	40	63,8	45,05
rCYL [D]	RE 2,0<br>LE 0,8	RE 2,4<br>LE 1,9	RE 5,4<br>LE 7,3	RE 2,6<br>LE 1,0	RE 2,1<br>LE 2,1	0,8	7,3	2,1
rAvgK [D]	RE 41,0<br>LE 40,5	RE 46,1<br>LE 64,4	RE 55,3<br>LE 49,2	RE 47,3<br>LE 45,7	RE 42,6<br>LE 42,2	40,5	64,4	45,9
rAA [%]	RE 94<br>LE 94,6	RE 91,4<br>LE 58,6	RE 100<br>LE 98,5	RE 94,5<br>LE 88,1	RE 90,7<br>LE 95,2	58,6	100	94,25
rACCP [D]	RE 41,0<br>LE 40,5	RE 46,5<br>LE 62,1	RE 55,1<br>LE 48,7	RE 47,8<br>LE 45,8	RE 42,6<br>LE 42,6	41	62,1	46,15
CAT[μm]	RE 539<br>LE 529	RE 606<br>LE 734	RE 573<br>LE 552	RE 480<br>LE 505	RE 545<br>LE 548	480	734	546,5
CTT [μm]	RE 520<br>LE 509	RE 528<br>LE 395	RE 356<br>LE 459	RE 462<br>LE 498	RE 540<br>LE 540	356	540	503,5
CAT-CTT	RE 10<br>LE 20	RE 78<br>LE 375	RE 217<br>LE 93	RE 18<br>LE 7	RE 5<br>LE 8	5	375	19
ACD [mm]	RE 2,97<br>LE 2,94	RE 2,27<br>LE 1,9	RE 2,86<br>LE 2,81	RE 3,03<br>LE 2,95	RE 3,02<br>LE 2,91	1,9	3,03	2,925
ESI [%]	RE 0<br>LE 0	RE 95<br>LE 95	RE 95<br>LE 50	RE 61<br>LE 7	RE 0<br>LE 0	0	95	28,5

BCVA, best corrected visual acuity; TBUT, tear break-up time; [k] keratometric; [p] posterior; [r] real; Ks, keratometry steep; Kf; keratometry flat; CYL, astigmatism, AvgK average keratometry; Ecc, eccentricity of the corneal curve; AA, area analyzed, ACCP, average central corneal power; CAT, corneal apex thickness; CTT, corneal thinnest thickness, ACD, anterior chamber depth, Esi, ectasia screening index.

Table S3. Fourier Indices results.

Parameter	P1	P2	P3	P4	P5	min	max	median
3 mm k Spherical	RE 42,11<br>LE 41,79	RE 47,67*<br>LE 68,43*	RE 55,64*<br>LE 49,38*	RE 49,64*<br>LE 46,93	RE 43,84<br>LE 43,97	41,79	68,43	47,3
3 mm k Reg. Astigmatism	RE 0,94<br>LE 0,21	RE 1,47*<br>LE 5,67*	RE 1,97*<br>LE 3,57*	RE 1,51*<br>LE 0,69	RE 1,19<br>LE 1,21	0,21	5,67	1,34
3 mm k Asymmetry	RE 0,41<br>LE 0,4	RE 7,66*<br>LE 5,36*	RE 4,06*<br>LE 0,83*	RE 2,64*<br>LE 0,67	RE 0,21<br>LE 0,14	0,14	7,66	0,75

3 mm k Higher Order	RE 0,28* LE 0,34*	RE 2,36* LE 5,94*	RE 6,69* LE 1,74*	RE 0,12 LE 0,11	RE 0,16 LE 0,8	0,11	6,69	0,34
6 mm k Spherical	RE 42,1 LE 41,83	RE 44,93 LE 64,18*	RE 53,55* LE 48,56*	RE 48,65* LE 46,74	RE 43,77 LE 43,6	41,83	64,18	45,84
**6 mm k Reg. Astigmatism**	**RE 1,08* LE 0,3**	**RE 1,84* LE 4,86***	**RE 2,14* LE 3,32***	**RE 1,48* LE 0,6**	**RE 1,21* LE 1,31***	**0,3**	**4,86**	**1,39**
6 mm k Asymmetry	RE 0,48 LE 0,5	RE 10,39* LE 5,81*	RE 6,07* LE 1,32*	RE 3,19* LE 0,9*	RE 0,38 LE 0,31	0,31	10,39	1,11
6 mm k Higher Order	RE 0,42* LE 0,39*	RE 2,32* LE 6,01*	RE 0,81* LE 1,72*	RE 0,15 LE 0,12	RE 0,16 LE 0,22	0,12	6,01	0,41
3 mm a Spherical	RE 46,92 LE 46,56	RE 53,11* LE 74,32*	RE 61,98* LE 55,01*	RE 54,2* LE 52,28	RE 48,84 LE 48,99	46,56	74,32	52,69
3 mm a Reg, Astigmatism	RE 1,05 LE 0,24	RE 1,64* LE 6,31*	RE 2,2* LE 3,97*	RE 1,64* LE 0,77	RE 1,33 LE 1,34	0,24	6,31	1,49
3 mm an Asymmetry	RE 0,46 LE 0,45	RE 8,53* LE 5,96*	RE 4,52* LE 0,93*	RE 3,55* LE 0,75*	RE 0,23 LE 0,16	0,16	8,53	0,84
3 mm a Higher Order	RE 0,31* LE 0,38*	RE 2,63* LE 6,61*	RE 0,77* LE 1,94*	RE 0,17 LE 0,12	RE 0,18 LE 0,20	0,12	6,61	0,34
6 mm a Spherical	RE 46,9 LE 46,6	RE 50,5 LE 71,5*	RE 59,66* LE 54,10*	RE 54,2* LE 52,08	RE 48,76 LE 48,57	46,6	71,5	51,29
**6 mm a Reg. Astigmatism**	**RE 1,2* LE 0,33**	**RE 2,06* LE 5,41***	**RE 2,38* LE 3,69***	**RE 1,64* LE 0,67**	**RE 1,35* LE 1,46***	**0,33**	**5,41**	**1,55**
6 mm an Asymmetry	RE 0,54 LE 0,56	RE 11,57* LE 7,59*	RE 6,76* LE 1,47*	RE 3,55* LE 1,01*	RE 0,42 LE 0,35	0,35	11,57	1,24
6 mm a Higher Order	RE 0,47* LE 0,43*	RE 2,59* LE 6,73*	RE 0,91* LE 1,92*	RE 0,17 LE 0,13	RE 0,18 LE 0,24	0,13	-6,73	0,45
3 mm p Spherical	RE -6,02 LE -6,15	RE -6,54 LE -10,2*	RE -7,02* LE -6,43	RE -7,38* LE -6,72	RE -6,4 LE -6,34	-10,2	-6,02	-6,48
**3 mm p Reg. Astigmatism**	**RE 0,14 LE 0,14**	**RE 0,38* LE 1,93***	**RE 0,88* LE 0,23**	**RE 0,34* LE 0,19**	**RE 0,35* LE 0,33**	**0,14**	**1,93**	**0,34**
**3 mm p Asymmetry**	**RE 0,12 LE 0,15***	**RE 1,25* LE 0,41***	**RE 1,97* LE 0,86***	**RE 1,09* LE 0,14***	**RE 0,07 LE 0,03**	**0,03**	**1,97**	**0,15**
**3 mm p Higher Order**	**RE 0,04* LE 0,06***	**RE 0,23* LE 1,81***	**RE 0,93* LE 0,16***	**RE 0,07* LE 0,03**	**RE 0,02 LE 0,03**	**0,02**	**1,81**	**0,06**

6 mm p Spherical	RE -6,0<br>LE -6,1	RE -6,43<br>LE -9,51*	RE -6,89*<br>LE -6,42	RE -7,14*<br>LE -6,69	RE -6,36<br>LE -6,3	-9,51	-6,0	-6,425
**6 mm p Reg. Astigmatism**	**RE 0,13<br>LE 0,12**	**RE 0,3<br>LE 1,74***	**RE 0,73*<br>LE 0,29**	**RE 0,34*<br>LE 0,18**	**RE 0,32*<br>LE 0,31***	**0,12**	**1,74**	**0,31**
6 mm p Asymmetry	RE 0,12<br>LE 0,14	RE 1,31*<br>LE 0,75*	RE 2,03*<br>LE 0,93*	RE 1,05*<br>LE 0,13	RE 0,09<br>LE 0,04	0,04	2,03	0,445
**6 mm p Higher Order**	**RE 0,05<br>LE 0,06***	**RE 0,21*<br>LE 1,81***	**RE 0,84*<br>LE 0,15***	**RE 0,06*<br>LE 0,03**	**RE 0,02<br>LE 0,04**	**0,02**	**1,81**	**0,06**

*out of reference range [normative database]; bolt font – parameters, which showed abnormalities for at least four patients in at least one eye; RE right eye; LE left eye; [k], keratometric; [a] anterior; [p] posterior; Reg., regular.
